# Supplementary material for: Learning curve and surgical outcome of robotic assisted colorectal surgery with ERAS program
Source: Sci Rep. 2022 Nov 29;12:20566. doi: 10.1038/s41598-022-24665-w (PMC9709162; doi:10.1038/s41598-022-24665-w)
Supplement: Supplementary file 2 — Supplementary Information 2. [file 41598_2022_24665_MOESM2_ESM.zip › RAL Raw data-Ñ[▒K/114/HS_ñw╝╨░O▒Kñσ.pdf]

正式摘要(電子簽章：000000226397189)

W83 / W83 ☐ 英文標題(注意:無英文姓名-請補病人基本檔!) ☐【讀取】憑證

臺中榮民總醫院

出院病歷摘要

轉入號 : 轉出號 :

[redacted]

**(8)**流水編號	
**(9)**入院日期	110/09/13 (2021)
**(10)**轉科日期	
**(11)**出院日期	110/10/02 (2021) 住院天數計 19 日

診斷

- 入院 :Rectal adenocarcinoma with liver metastasis, cT3N2M1
- 出院 :Rectal adenocarcinoma with liver metastasis, cT3N2M1 s/p robotic-assisted low anterior resection with pelvic lymph node dissection and laparoscopic S2,4,7 hepatectomy on 2021/09/25, pT3N1aM1a

主訴

- bloody stool for 5 months

病史

- This 45-year-old woman with history of s/p caesarean section and denying systemic disease was suffered from intermittent bloody stool for 5 months, she also mentioned poor appetite and body weight loss recently. She had initial medical visit to 成大斗六分院 and abdominal CT showed rectal tumor with liver metastasis, cT3N2M1. As above problems, she was admitted for further care.

體檢發現

-
  - General appearance :
    - easy looking , conscious clear , without cardiopulmonary distress
    - Vital signs自動帶入date 體溫(BT) 脈搏(P) 呼吸(R) 血壓(BP) 身高(BH) 體重(BW) BMI
      20210913 36.6℃ 90次/分 17次/分 158/84mmHg 156.8cm 51kg 20.7
  - HEENT :
    - Eyes: no anemic conjunctiva, no icteric sclera, normal EOM
    - ENT: no otorrhea, no rhinorrhea, no erythematous mucosa, no injected tonsil, no deformity
  - Neck: supple, freely movable, trachea midline,no deformity, no engorged jugular vein , no palpable thyroid or lymph node enlargement
  - Chest:
    - Inspection: Grossly normal, symmetric and fully expansion, no accessory muscle used, no operation scar, no spider angiomata, no gynecomastia
    - Palpation: no palpable mass or nodule, no subcutaneous emphysema, no tenderness
    - Ausculation: clear breathing sound
  - Heart:
    - Percussion: normal heart size
    - Ausculation: regular heart beat, normal S1, S2, no gallop, no murmur
  - Abdomen:
    - Inspection: in
      - flat contour, symetrically, no scar, no stria, no dilated veins, no rashes
    - Ausculation: normoactive bowel sound, no abdominal bruits
    - palpation: soft, no muscle guarding or rigidity, no local tenderness, no rebounding pain, no palpable mass, no palpable pulsation
    - Percussion: no shifting dulllness, no hepatomegaly, no splenomagaly
-

Peripheral pulsation :	CA	BA	RA	FA	PA	PTA	DPA
Right side :	++	++	++	++	++	++	++/+++

- | Left side :|++|++|++|++|++| ++ |++/+++|
    - Genito-anus: no tenderness or palpable mass lesion or rectal shelf, normal external genitalia, normal anal tone, brown stool

CRS CONDITION

-
    - Lung

手術日期及方法

- 手術日期及方法20210925 1.LIVER PARTIAL HEPATECTOMY2.Biliary cholecystectomy3.Laparoscopy4.
  20210925 Robot Assisted Low Anterior Resection
  ILEOSTOMY

住院治療經過

- After admission, we performed colonoscopy for rectal tumor biopsy on 2021/09/14. We had consult GS doctor for surgical evaluation of hepatectomy. She started the short-course radiotherapy for 5 times (9/17-9/24). We had explained surgical indication and risk for patient. She underwent robotic-assisted low anterior resection with pelvic lymph node dissection and laparoscopic S2,4,7 hepatectomy smoothly on 2021/09/25. After surgery, we let her try diet on POD1 as her tolerance. Infrahepatic drain was removed on POD4. CVC and pelvic drain was removed on POD6. Under the stable condition, she was discharged on 10/2.

合併症

- nil

一般檢查

- 累積報告 :

DATE	TIME	ALB	TP	BIL.T	BIL.D	ALKP	AST	ALT	LDH	AMY	LIPASE	NH3	Ferritin	UA
1100913	1645	4.0		0.15	0.05	69	18	10	253					
1100926	0031	2.9	4.9	0.49	0.15	55	552	393						
1100927	0956	2.8		0.46	0.16	60	180	216						
1100930	0849	3.0		0.35	0.13	140	95	115						

DATE	TIME	WBC	RBC	HGB	HCT	MCV	PLT	BLST	NEUT	BAND	SEG	LYM	MON	EOS	BAS
1100913	1645	8630	3.91	10.8	34.2	87.5	442		67.2			24.8	7.0	0.7	0.3
1100926	0023	6100	3.13	8.7	27.1		221		91.6			2.6	5.6	0.0	0.2
1100927	0913	7250		8.7			168		89.6			5.8	4.4	0.1	0.1
1100930	0919	4360		9.4	28.8		166		78.9			11.9	7.6	1.1	0.5

DATE	TIME	NA	K	CL	BUN	CREAT	eGFR	CA	Mg	P	CRP	ESR	PCT	D-Dimer	LACTATE
1100913	1645	138	4.0	104	8	0.58		8.3							
1100926	0031	147	2.9	110	8	0.54	129.76	7.6							
1100927	0956	140	3.8		8	0.52			1.7						
1100930	0849	138	3.8		4	0.48	148.65								

DATE	TIME	GLUCOSE	HbA1C	CHOL	TG	HDL	LDL	KETONE	CK	CKMB	TRO-T	PT-P	APTT-P	NT-pro BNP
1100913	1645	112										9.8	24.5	
1100913	2234		5.6											

特殊檢查

病理報告

- 活體組織切片病理報告　手術日期：**110/09/25**　病理號：**S1030348**

  診斷醫師:王任卿 報告日期:110/09/30
  Pathologic diagnosis:
  1. Liver, S2, hepatectomy --- Metastatic adenocarcinoma, moderately differentiated, consistent with colorectal origin. The resection margin is close to the tumor (less than 1 mm).
  2. Liver, S4, hepatectomy --- Metastatic adenocarcinoma, moderately differentiated, consistent with colorectal origin. The resection margin is close to the tumor (less than 1 mm).
  3. Liver, S7, hepatectomy --- Metastatic adenocarcinoma, moderately differentiated, consistent with colorectal origin. The resection margin is involved by the tumor.
  4. Gallbladder, cholecystectomy --- Autolysis.
  5. IMA root, dissection --- Unremarkable fibrovascular tissue and lymph node (0/1).
  6. Rectum, cut end, distal, resection --- Chronic inflammation.

- 7. Rectum, robot low anterior resection --- a. Adenocarcinoma, moderately differentiated. Tumor invaded to perirectal soft tissue and metastasized to perirectal lymph node (1/10). The bilateral cut ends are free of tumor. b. Endometriosis of perirectal soft tissue.
  8. Lymph node, lateral, dissection --- Unremarkable (0/3).
  9. "Lymph node", pelvic, dissection --- Unremarkable fibrovascular tissue.
  Ancillary study for diagnosis:
  IHC stains for PMS2 and MSH6 are done on section E. Both above markers are preserved.
  Prognostic and predictive factor:
  1. Tumor size: 5x3.9 cm.
  2. Depth of invasion: Tumor invades to pericolorectal tissue.
  3. Histologic grade: G2: Moderately differentiated.
  4. Angiolymphatic invasion: Not identified.
  5. Perineural invasion: Not identified.
  6. Tumor deposits (discontinuous extramural extension): Not identified.
  7. Macroscopic tumor perforation: Not identified.
  8. Surgical Margins: a) Proximal Margin: Uninvolved by invasive carcinoma, distance of tumor from margin: 7.5 cm. b) Distal Margin: Uninvolved by invasive carcinoma, distance of tumor from margin: 1.8 cm. c) Radial or Mesenteric Margin: Uninvolved by invasive carcinoma, distance of tumor from margin: 3 mm.
  9. Lymph node status: Involved (1/14).
  10. Extranodal involvement: Present.
  11. Tumor regression grading S/P CCRT: No known presurgical therapy.
  12. Type of polyp in which invasive carcinoma arose: Not identified.
  13. Additional pathologic findings: Not identified.
  14. Pathological TNM Stage: pT3N1aM1a(According to the eighth edition, American Joint Committee on Cancer Staging Guidelines for Tumors).
  15. TNM descriptors: Not applicable.
  Gross description:
  The specimen consists of 1) a piece of liver tissue, less than 10 gm and 1.1x0.8x0.3 cm, labeled as "liver S2". A well defined yellow green solid nodular mass, 0.8 cm, is noted close to the resection margin. 2) a piece of liver tissue, 60 gm and 7x5.3x3.6 cm, labeled as "liver S4". Two well defined yellow green solid nodular masses, up to 4.2 cm, are noted close to the resection margin. 3) a piece of liver tissue, 30 gm and 5.2x4x2.1 cm, labeled as "liver S7". A well defined yellow green solid nodular mass, 2.3 cm, is noted close to the resection margin. 4) a piece of gray green opened gallbladder, 5.5x2.2x0.7 cm. Neither stone nor tumor found grossly. 5) IMA root, a piece of tan yellow soft tissue, 3x2.7x0.6 cm. 6) distal cut end, a piece of tan mucosa-covered soft tissue, 1.8x1.6x0.4 cm. 7) a segment of colon, 15 cm in length and 4.5 cm in proximal cut end circumference, 6 cm in distal circumference, and perirectal soft tissue. A gray tan firm annular elevated and centrally ulcerative tumor, 5x3.9 cm, is noted, 1.8 cm to the nearest cut end. On cross section, the tumor invades to the mesocolic soft tissue with 3 mm to the radial margin, and serosal retraction is also noted. The both cut ends are free of tumor grossly. 8) "lateral lymph node", a piece, 3.3x2.1x0.9 cm. 9) "pelvic lymph node", a piece, 3x2x0.4 cm.
  Representative part for section: A) proximal cut end B) distal cut end C-E) tumor F) colon G) liver S2 H) liver S4 J) liver S7 K-M) specimen 4-6 N-P) specimen 8-9 Q-Z) lymph nodes.
  Note:
  1. The interpretation of MMR panel and recommended studies are as below: (1) No loss of nuclear expression of MMR proteins: low probability of microsatellite instability-high (MSI-H) . (2) Loss of nuclear expression of MLH1 and PMS2: testing for methylation of the MLH1 promoter and/or mutation of BRAF is indicated (the presence of a BRAF V600E mutation and/or MLH1 methylation suggests that the tumor is sporadic and germline evaluation is probably not indicated; absence of both MLH1 methylation and of BRAF V600E mutation suggests the possibility of Lynch syndrome, and sequencing and/or large deletion/duplication testing of germline MLH1 may be indicated). (3) Loss of nuclear expression of MSH2 and MSH6: high probability of Lynch syndrome (sequencing and/or large deletion/duplication testing of germline MSH2 may be indicated, and, if negative, sequencing

- and/or large deletion/duplication testing of germline MSH6 may be indicated). (4) Loss of nuclear expression of MSH6 only: high probability of Lynch syndrome (sequencing and/or large deletion/duplication testing of germline MSH6 may be indicated). (5) Loss of nuclear expression of PMS2 only: high probability of Lynch syndrome (sequencing and/or large deletion/duplication testing of germline PMS2 may be indicated). (6) Please note: There are exceptions to the above IHC interpretations. These results should not be considered in isolation, and clinical correlation with genetic counseling is recommended to assess the need for germline testing.
  2. This case has been peer reviewed by two doctors.
  #T-59600_2 #M-81403_2 2 1105 S0000F
  #T-C4560_2 #M-81406_2 2 1105 000000
  #T-62000_2 #M-81406_2 2 1100 000000
  #T-63000_2 #M-09413_2 0 1100 000000

其它

出院時情況

- 3.改門診治療

出院指示

藥名	劑量	劑量單位	用法	頻次	數量	數量單位	現況	天數	單位
(口)Takepron OD tab 30mg	1	TAB	PO	QDAC	11	TAB	TH	11	天
Ultracet tab	1	TAB	PO	Q6H	44	TAB	TH	11	天
Anxiedin tab 0.5mg	1	TAB	PO	QN	11	TAB	TH	11	天

出院計劃

主治醫師 2062F 蔣鋒帆 / 住院醫師 2331F 陳凱祥

正式摘要(電子簽章：000000226397189)

W83 / W83

doctorRank:B
